# Novel fixed *z*-direction (FiZD) kidney primordia and an organoid culture system for time-lapse confocal imaging

**DOI:** 10.1242/dev.142950

**Published:** 2017-03-15

**Authors:** Ulla Saarela, Saad Ullah Akram, Audrey Desgrange, Aleksandra Rak-Raszewska, Jingdong Shan, Silvia Cereghini, Veli-Pekka Ronkainen, Janne Heikkilä, Ilya Skovorodkin, Seppo J. Vainio

**Affiliations:** 1 Faculty of Biochemistry and Molecular Medicine, University of Oulu, 90220 Oulu, Finland; 2 Laboratory of Developmental Biology, Biocenter Oulu and InfoTech, 90220 Oulu, Finland; 3 Department of Medical Biochemistry and Molecular Medicine, Oulu Center for Cell Matrix Research, 90220 Oulu, Finland; 4 Center for Machine Vision Research, Department of Computer Science and Engineering, University of Oulu, 90014 Oulu, Finland; 5 Sorbonne Universités, UPMC Univ Paris 06, IBPS – UMR7622 Developmental Biology, Paris F-75005, France; 6 Institut de Biologie Paris-Seine (IBPS) – CNRSUMR7622 Developmental Biology, F-75005 Paris, France; 7 Biocenter Oulu, University of Oulu, 90220 Oulu, Finland

**Keywords:** Kidney, Organ culture, Time-lapse, Organoid, Imaging

## Abstract

Tissue, organ and organoid cultures provide suitable models for developmental studies, but our understanding of how the organs are assembled at the single-cell level still remains unclear. We describe here a novel fixed *z*-direction (FiZD) culture setup that permits high-resolution confocal imaging of organoids and embryonic tissues. In a FiZD culture a permeable membrane compresses the tissues onto a glass coverslip and the spacers adjust the thickness, enabling the tissue to grow for up to 12 days. Thus, the kidney rudiment and the organoids can adjust to the limited *z*-directional space and yet advance the process of kidney morphogenesis, enabling long-term time-lapse and high-resolution confocal imaging. As the data quality achieved was sufficient for computer-assisted cell segmentation and analysis, the method can be used for studying morphogenesis *ex vivo* at the level of the single constituent cells of a complex mammalian organogenesis model system.

## Abstract (precis)

**Summary:** Time-lapse confocal imaging of organoids and embryonic tissues through fixed *z*-direction culture allows long-term single-cell resolution live imaging of tissue growth and morphogenesis.

## INTRODUCTION

High-resolution confocal imaging is an important means of studying the cellular behavioural dynamics of organ morphogenesis, a process that involves division, migration, death and changes in shape of the cells. Yet, we have a poor understanding of how these cellular events establish the shapes of the eventual organs. The rapid development of microscopic imaging (Huisken et al., 2004) and automated image analysis technologies (Eliceiri et al., 2012) has provided the basis for achieving a better understanding of these processes, as has been shown in certain model organisms (Khan et al., 2014).

In the traditionally used Trowell setup, the explant is placed on a filter supported by a metal grid (Auerbach and Grobstein, 1958; Grobstein, 1955; Kispert et al., 1998; reviewed in Rak-Raszewska et al., 2015; Saxén, 1987), and more recently, commercial inserts have also been used for this purpose (Costantini et al., 2011). For high-resolution time-lapse imaging, however, more sophisticated ways of organizing the organotypic culture experiments using an on-stage microscope incubator are required. The branching morphogenesis and patterning of the ureteric bud (UB) has been monitored using Transwell inserts (Watanabe and Costantini 2004) and the low-volume method (Lindström et al., 2014), and there have been reports on UB cell behaviour (Packard et al., 2013) and the movement of progenitor cells during UB branching morphogenesis (Riccio et al., 2016) based on the use of Transwell inserts. The same system was used to investigate the effect of β-catenin levels during nephron patterning (Lindström et al., 2015) and for tracking the movements of cap mesenchymal cells (Combes et al., 2016). In all of these applications, however, the imaging of individual cells was limited, hindering the full use of these elegant models.

We report here a novel technique for culturing embryonic kidneys and organoids in a fixed *z*-direction culture (FiZD) set up in a restricted space between a Transwell filter and a glass coverslip. This technology provides high-quality single-cell-resolution time-lapse imaging and permits computer-assisted analysis of the assembly of complex cellular structures.

## RESULTS AND DISCUSSION

**Fig. 1.**
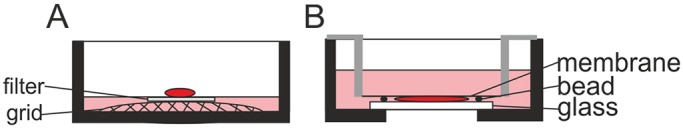
**Fixed *z*-direction system.** (A) In the Trowell system the explant is placed on a filter supported by a grid holding the explant at the air-liquid interface. (B) In FiZD culture the explant is between a glass surface and a Transwell insert. Spacer beads are used to adjust the thickness of the tissue.

The main disadvantage of traditional culture methods for conducting time-lapse imaging is poor light microscopy image quality. The reasons for this are the long focal length, the thickness of the tissue, and the air-liquid interface or Transwell membrane between the specimen and the objective (Fig. 1A).

**Fig. 2.**
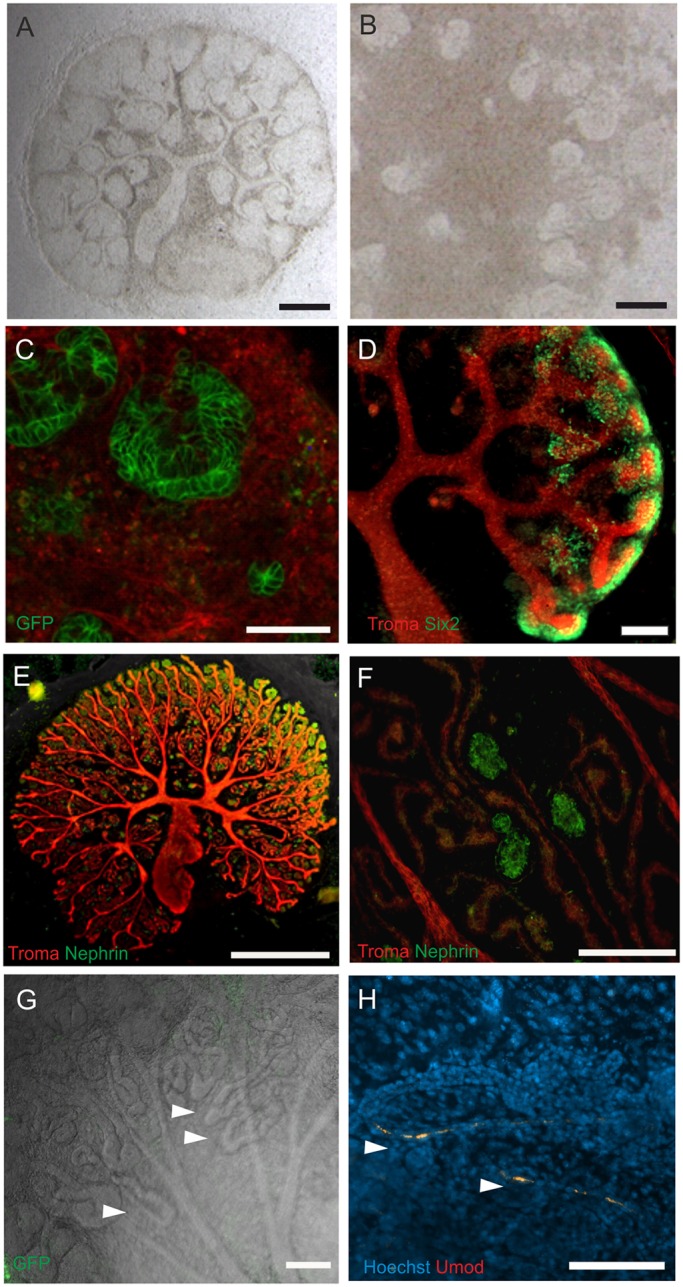
**Kidney morphogenesis progresses well in FiZD culture.** Embryonic kidneys (A,D-G) and organoids (B,C,H) were grown in FiZD culture. (A) Brightfield micrograph of an intact embryonic kidney, and (B) kidney organoid cultured for 7 days. (C) Snapshot of the time-lapse image stack depicting *Wnt4Cre*-activated GFP expression in the assembling nephrons on the fourth day of FiZD culture. (D) Six2 and Troma-I (Krt8) staining highlight nephron precursors and UB bifurcations, respectively, in a 7-day FiZD culture. (E) Kidney rudiment FiZD-cultured for 12 days. Troma-I and Nephrin staining depict the UB bifurcations and podocytes, respectively. (F) High-power magnification of the Nephrin+ podocytes and Troma-I+ UB in E. (G) Frame from the time-lapse image stack of *Flk-GFP* endothelial cells. Henle's loop-like structures (arrowheads). (H) Umod+ loops of Henle (arrowheads) in an organoid cultured for 7 days. Umod and Hoechst stainings. Scale bars: 100 µm in A-D,F-H; 1000 µm in E.

This led us to consider whether restricting the ability of the sample to grow in the *z*-direction by use of a porous membrane (Fig. 1B) would enable better imaging quality. This proved to be optimal for supporting organogenesis and providing a high imaging quality (Fig. 2).

The *z*-direction was regulated by polyester beads that serve as spacers (Fig. 1B). We found empirically that a spacer of <20 µm diameter did not prevent mechanical destruction of the samples (Fig. S2A-F) and 40 µm beads gave greater variation in sample viability (Fig. S2G-L), whereas 70 µm beads provided the best conditions for both high-quality imaging and optimal culture conditions (Fig. S2M-R). These observations indicate that the diameter of the beads must be optimized for each experiment according to the size and properties of the sample.

The formation of the renal vesicle, comma-shaped and S-shape bodies, and eventually segmented nephrons with Bowman's capsule and loops of Henle were also observed in intact kidneys and organoids in FiZD culture (Fig. 2A-H; Movies 1-3,6-10).

We addressed the question of how renal vasculature structures labelled with GFP reporter emerge in the FiZD setup of intact kidney (Movie 1), which was in line with previously published data (Halt et al., 2016).

We employed mT/mG;HoxB7Cre transgenic mice to study the degree of UB development, as highlighted by the *HoxB7Cre*-activated *GFP* expression in the epithelium of the UB (Movie 2).

FiZD was also used for studying nephron development in mT/mG;Wnt4Cre organoids. The metanephric mesenchyme was induced to undergo nephrogenesis by transient exposure to a GSK-3α/β inhibitor. In such a setting the behaviour of individual GFP+ cells could be monitored in the images captured, allowing the analysis of cell movements and nephron patterning (Movie 3).

**Fig. 3.**
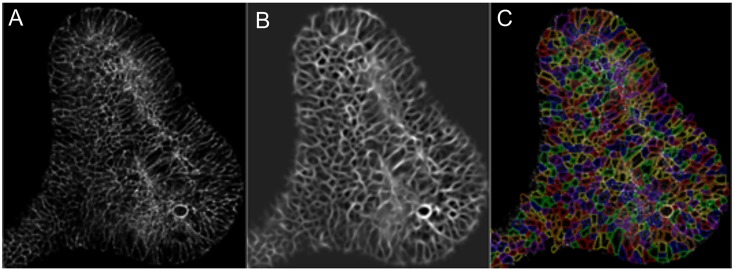
**FiZD culture-generated image data stacks can be subjected to computer-assisted cell segmentation.** Time-lapse images were captured at 5 min intervals and processed with a program developed in Matlab. (A) Frame of the 3D image stack. (B) Ridge-enhanced image after deconvolution. (C) Cell boundaries highlighted by the cell segmentation program.

We next explored whether the data would allow computer-assisted cell segmentation. Confocal microscopic micrographs of GFP+ UB cells before (Fig. 3A) and after deconvolution (Fig. 3B) to improve the image resolution enabled cell segmentation (Fig. 3C). The GFP allowed tracking of the UB cell membranes as a result of the *HoxB7Cre*-mediated *GFP* activation (Movie 4).

**Fig. 4.**
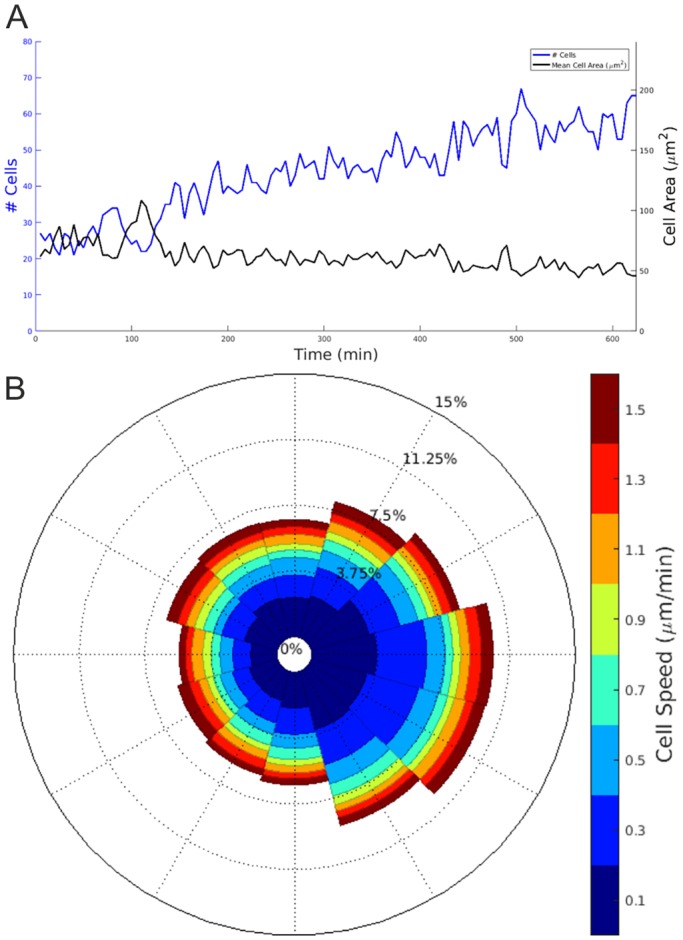
**Segmented FiZD culture-based image data enable analysis of kidney morphogenetic parameters.** (A) Increase in the number of UB tip cells in the right-hand side of the growing kidney (blue), and the mean cell area (black). (B) Wind rose plot illustrating the direction of UB tip cell migration. The lengths of the spokes of the wind rose plot indicate the proportions of cells moving in a given direction and the thicknesses of the colour bands within a spoke indicate their speed distribution. Both wind rose and cell count plots show that the kidney is growing towards the right, as the number of cells in the right half is increasing and more cells are moving towards the right. The data correspond to the single *z*-projection presented in Movie 3.

The computer-assisted image analysis enabled several key morphogenetic parameters to be examined simultaneously (Fig. 4A,B). The data derived from the FiZD served to identify the speed and direction of UB cell migration as presented in a wind rose plot (Movie 5), as used in certain systems (Stegmaier et al., 2016). By analysing segmentation data it is possible to study cellular behavioural dynamics in detail during morphogenesis, including the processes that take place during the development of a nephron.

The low-volume method provided better quality images than Trowell culture (Sebinger et al., 2010) and was compared with the FiZD method. The FiZD and low-volume experiments were organized in one 6-well plate, with confocal imaging of intact kidney cultures and organoids (Fig. S3, Movies 6,7,10). To provide an objective comparison, the culture medium was changed daily in both types of culture, even though this is not required for the FiZD system. The results demonstrate the benefits of the FiZD system: better overall imaging quality, easy medium change without affecting the sample, reduced thickness of the sample and perfect stability of the image. Additionally, several samples could be assembled simultaneously in one FiZD setup with precise control of their position, whereas the low-volume method does not allow more than one sample per silicon chamber (note the fusion of what were initially two organoids in Movie 10, right panel). The FiZD method was also compared with the Transwell culture method (Movie 11). However, due to the low signal the laser power had to be doubled and this was toxic to the kidney rudiment (Fig. S4). To present the FiZD time-lapse data at the highest possible resolution, we made a short time-lapse of the developing nephrons (Movie 8), 3D structural analysis of the same developing nephron was provided by displaying all the *z*-layers at one point in time (Movie 9).

The capacity of the kidney to develop under FiZD culture conditions could be attributed to the conditions under which this occurs during normal nephrogenesis, when the nephron takes shape during its assembly. It has been shown that the viscoelasticity of the tissue, and thus also its rigidity, depends on the composition and crosslinking of the extracellular matrix components and their binding to cells (Forgacs et al., 1998; Phillips and Steinberg, 1978).

As judged by several organogenesis indicators presented here, kidney morphogenesis advances well under FiZD conditions, and at the same time the FiZD setup provides a superior capacity for image quality due to the reduction in tissue thickness. Here, multiple tissue samples can be cultured simultaneously and organs can be fixed in defined positions prior to culture. The culture medium can be changed or supplemented with given factors in a manner that does not disturb the development of the tissue or its capacity to retain its initial position. Moreover, the relatively large volume of the wells and lower laser power enables long-term imaging. Most importantly, the FiZD is well suited for microscope stage incubation system culture and provides an excellent platform for high-resolution confocal imaging, automatic cell segmentation, tracking and image quantification that would also be suitable for use with other organotypic cultures (Prunskaite-Hyyryläinen et al., 2016) and organoids.

## MATERIALS AND METHODS

### Mouse models and dissection of kidneys

The embryonic kidneys were dissected from wild-type CD-1 embryos or crosses between *Tie1Cre* (Gustafsson et al., 2001), *Hoxb7Cre* (Yu et al., 2002), *Wnt4Cre* (Shan et al., 2010), *Flk1-GFP* (Licht et al., 2004), *GFP* (Hadjantonakis et al., 1998) or *tomato floxed Rosa26 Green fluorescent protein (GFP)* (*mT/mG*) reporter mice (Muzumdar et al., 2007), as described by Junttila et al. (2015). Animal care and procedures were in accordance with Finnish national legislation for the use of laboratory animals, the European convention for the protection of vertebrate animals (ETS 123) and EU Directive 86/609/EEC.

### Embryonic kidney organoids

An organoid can be defined as an *in vitro* 3D aggregate derived from primary tissue (Auerbach and Grobstein, 1958; Junttila et al., 2015; Unbekandt and Davies, 2010; Vainio et al., 1992), embryonic stem cells (Eiraku et al., 2008) or induced pluripotent stem cells (iPSCs) (Morizane et al., 2015; Takasato et al., 2015), which are capable of self-renewal and self-organization, and exhibit similar organ functionalities as the tissue of origin (Fatehullah et al., 2016). In our experiments we used organoids derived from primary cell cultures isolated from embryonic metanephric mesenchyme, as described by Junttila et al. (2015), except that 10 µM BIO (6-bromoindirubin-3′-oxime, Sigma) was used for induction.

### Embryonic kidney organoids derived from frozen primary cells

The organoids in the experiments presented in Fig. 2H and Movie 10 were made using frozen primary cells. Dissociated embryonic day (E)11.5 mesenchyme or intact E13.5 mT/mG kidneys were suspended in 20% DMSO/80% FBS and frozen in cell-freezing containers at −80°C and transferred the next day to liquid nitrogen. Upon usage the cell vial was quickly warmed, the cells washed twice with medium and then pelleted (4 min at 1380 ***g***) to make organoids, as in the usual protocol. The intact kidneys were dissociated during the freezing/thawing process. When whole kidneys were used the cell suspension included UB cells, which induced the nephrogenesis.

### Assembly of the tissue culture

The FiZD conditions were set up in 6-well CellStar plates (Greiner Bio-One) (Fig. S1). A hole (20 mm diameter) was drilled at the bottom of wells and glass coverslips (24×24 mm) were glued to the upper side of the bottom with either dental wax or Histoacryl glue (Braun, 1050052). The coverslips were cleaned with ethanol and sonication to promote affixation of the organ rudiments to the coverslip glass (supplementary Materials and Methods). The culture plates were rinsed with ethanol, distilled water and dried in a UV hood. Kidney organoids incubated overnight in a microfuge tube or E11.5 to E13.5 embryonic kidneys were arranged on the lower side of the Transwell insert membrane (Corning, 3450) and the excess volume of PBS was aspirated. Polystyrene beads (Corpuscular, 100263-10) were mixed with Matrigel on ice and a few microliters were added to the samples with care not to disturb their position on the filter. Matrigel can aid fixation of the specimen in a defined position, but it is not required (see Fig. 2B,D with Matrigel, and Fig. 2H without) and it did not influence morphogenesis. Next, the insert was turned so that the samples were face down, positioned in a well, and pressed gently into place. The rim of the insert was melted with a heated glass capillary at three points to fix the insert to the plate (Fig. S1). The well was filled with 2 ml DMEM (Gibco, 41965-039), 10% foetal calf serum (Gibco, 10500-064) and 1% penicillin/streptomycin (Sigma, P4333) and the plate was kept at 37°C and 5% CO_2_. The culture setups were repeated a minimum of five times for each type of experiment. For the low-volume culture system we followed the method described in Sebinger et al. (2010), repeating the experiment 15 times. For imaging of the kidney cultures on the top of the filter (Costantini et al., 2011) the standard 6-well glass-bottomed plates were used (BD Falcon).

### Time-lapse image capture

The culture plates were inserted into an on-stage incubator on a Zeiss LSM780 confocal microscope at 37°C and 5% CO_2_. The time-lapse images were captured at 5-20 min intervals and processed with ZEN 2012 (blue edition) (Zeiss), Huygens Professional (Scientific Volume Imaging) and Fiji (Schindelin et al., 2012).

### Whole mount immunostaining

The explants were transferred to 4% PFA for 20 min, washed in PBS, and stored at 4°C. For immunostaining, the samples were blocked for 1 h in 0.1% Triton-X100/1% BSA/10% goat serum/0.02 M glycine PBS at room temperature. Primary antibodies against Krt8 (1:100; clone Troma-I, AB_531826, Developmental Studies Hybridoma Bank), Nephrin (also known as Nphs1) (1:1000; a gift from Prof. Karl Tryggvason, Duke-NUS Medical School, Singapore), Six2 (1:200; 11560, Proteintech), Umod (1:20; LS-C150268, LSBio) and Pax2 (1:100; PRB-276P, Covance) were used for immunostaining.

After an overnight incubation at 4°C the samples were washed six times for 30 min in PBS and goat anti-rat Alexa Fluor 546 (1:1000; A11081, Molecular Probes) and goat anti-rabbit Alexa Fluor 488 (1:1000; A11008, Molecular Probes) or donkey anti-sheep NorthernLights NL557 (1:200; NL010, R&D Systems) were incubated 1 h at room temperature and washed several times with PBS. A Zeiss LSM780 microscope and Zeiss Axiolab were used for analysis and image capture.

### Automatic image analysis

Embryonic UBs labelled with *HoxB7Cre*-activated GFP, highlighting the cell boundaries, were segmented and tracked using a program developed in Matlab (MathWorks). Hessian ridge enhancement (Hodneland et al., 2009) was used to enhance the cell membrane intensity, fill the membrane gaps and remove cytoplasmic fluorescence. H-minima transformation (Soille, 1999) was used to filter out local minima and the Watershed transformation (Meyer, 1994) for cell segmentation. The centroids of the segmented cells in the first frame were used to initialize the cell tracks and the Hungarian algorithm (Munkres, 1957) was used to associate detections with tracks. For details on segmentation of image data and preparation of wind rose plots, see the supplementary Materials and Methods.
